# Effectiveness of Omega-3 polyunsaturated fatty acids reducing severe symptoms in patients diagnosed with Borderline Personality Disorder (BPD)

**DOI:** 10.1192/j.eurpsy.2023.683

**Published:** 2023-07-19

**Authors:** T. Gutierrez Higueras, F. Calera Cortés, L. Montes Arjona, S. Vicent Fores, S. Sainz de la Cuesta Alonso, E. D. Servín López

**Affiliations:** Psychiatry, Hospital Universitario Reina Sofía, Córdoba, Spain

## Abstract

**Introduction:**

Omega-3 polyunsaturated fatty acids (PUFAs) have been studied in relation to mental illness. Among the most important omega 3 fatty acids, eicosapentaenoic acid (EPA) and docosahexaenoic acid (DHA) stand out, both derived from alpha-linolenic acid. Both EPA and DHA are essential fatty acids. Consequently, mammals are not capable of synthesizing them and must incorporate them through the consumption of products such as fish oil. The interest about the role of omega 3 fatty acids for the treatment of patients with impulsiveness, hostility and aggressiveness is growing and originated from the finding of a low level of EPA and DHA in the central nervous system of these individuals.

**Objectives:**

To determine the evidence on the effectiveness of omega-3 acids in reducing severe symptoms in patients diagnosed with Borderline Personality Disorder.

**Methods:**

A literature review was carried out in Epistemonikos, using the descriptors: “borderline personality disorder” AND “Omega-3”. 7 results are obtained. The results of a time limit of 10 years with meta-analyses and systematic reviews were filtered, obtaining 7 results and selecting 3 of them for their relevance to the PICO question. Subsequently, the search was repeated using the same descriptors and time limit in the Cochrane Library, NICE, and Pubmed; no selection was made by coincidence of those previously selected.

**Results:**

The first systematic review studied the effectiveness of omega-3 fatty acids in symptomatology associated with BPD, with differentiation of the domains of affective, impulsive and cognitive-perceptual symptoms. Within the meta-analysis, 5 randomized controlled trials (RCTs) were included that compared omega-3 fatty acids with placebo or any active comparator, four of these RCTs verified the effect of omega-3 acids in 137 patients with BPD or behavior related to the BPD.

The second systematic review, conducted in the Cochrane Collaboration, performed a meta-analysis of randomized comparisons of drug versus placebo. Twenty-seven trials testing first- and second-generation antipsychotics, mood stabilizers, antidepressants, and omega-3 fatty acids were included. For supplemental omega-3 fatty acids, significant effects were found in one study (n = 49 ) for reduction in suicidality (RR = 0.52, 95% CI 0.28 to 0.95) and depressive symptoms (RR = 0.48, 95% CI 0.28 to 0, 81).

**Conclusions:**

Available data indicate that marine omega-3 fatty acids improve BPD symptoms, particularly impulsive behavioral dyscontrol and affective dysregulation, reducing depressive symptoms and suicidal tendencies. Marine omega-3 fatty acids could be considered as a complementary therapy for the improvement of severe symptoms associated with patients with BPD.

**Disclosure of Interest:**

None Declared

